# Design and Development of a Machine Learning Model for Predicting ICU Patients’ Length of Stay

**DOI:** 10.7759/cureus.89903

**Published:** 2025-08-12

**Authors:** Dimitrios Kosmidis, Dimitrios Simopoulos, Nestoras Kosmidis, George Anastassopoulos

**Affiliations:** 1 Department of Nursing, Democritus University of Thrace, Greece, Alexandroupolis, GRC; 2 Department of Medicine, Democritus University of Thrace, Alexandroupolis, GRC; 3 Department of Informatics, Ionian University, Corfu, GRC

**Keywords:** ensemble learning, intensive care, length of stay, machine learning, nursing

## Abstract

Background and aim

Predicting the Length of Stay (LOS) for patients in the Intensive Care Unit (ICU) can aid in improving care management and resource allocation. Compared to traditional scoring systems, machine learning methods usually provide more accurate LOS predictions, highlighting the need for improved precision. This study aims to introduce and assess the stacked ensemble machine learning model capabilities to predict ICU LOS for both short- and long-term patient groups, using Acute Physiology and Chronic Health Evaluation (APACHE) IV-derived features.

Methods

We used approximately 148,000 patient records from the eICU Collaborative Research Database. To predict patient LOS, we first divided the patients into two groups (short- and long-term), based on their actual ICU LOS. Subsequently, we developed two stacked ensemble learning models for each patient group.

Results

For short-term patients, the Mean Absolute Error (MAE) was 1.037, whereas for long-term patients it was 1.997. Particularly, for patients with long-term actual ICU LOS (median 10.6 days), the respective predictions were clinically acceptable, suggesting the model’s dynamics in real ICU environment applications. The clinical utility of these predictions can help clinicians in managing patient care in the ICU. Validation of these findings on external datasets may increase the applicability in daily clinical practice. Limitations include the use of data only from United States ICUs, selection of input features, and lack of external validation, which may affect the further generalizability and applicability of our model to different clinical settings.

Conclusion

The clinical utility of these predictions can help clinicians in managing patient care in the ICU. Future validation of these findings on external datasets may increase the applicability in daily clinical practice.

## Introduction

Intensive care is an example of the balance required between increasing demand and limited supply of available beds that pressures health professionals to find Intensive Care Unit (ICU) services with shorter Length of Stay (LOS) and higher survival rates [1]. LOS and mortality in the ICU are two of the most used indicators - as a process or outcome - to assess the quality and efficiency of care [2-4]. LOS in the ICU has been also proposed as either an indirect or a direct outcome measure for quality of nursing care [5,6]. Various models, such as Acute Physiology and Chronic Health Evaluation (APACHE) and Sequential Organ Failure Assessment (SOFA), generally provide useful predictions of LOS, with good calibration [7]. However, these traditional models mainly focus on clinical characteristics based on specific fixed severity coefficients that do not fully reflect the ever-changing conditions of patients in the ICU. Limitations, such as their static chronological nature, dependence on static, pre-defined variables, inability to integrate data in real-time, and continuous recalibration needs to maintain their accuracy and reliability, have been noted [8].

Machine Learning (ML) is considered a branch of Artificial Intelligence (AI) that develops and evaluates algorithms. These algorithms are designed to train and "learn" from existing data and predict the results of new observations [9]. Predicted LOS using ML techniques is an innovative tool that has been shown to allow more accurate predictions and therefore safer use [10]. The major advantage in using ML is its dynamic nature, i.e., it can evaluate and adjust many parameters simultaneously even in real time [11]. Several studies have shown that the developed ML models deliver better efficiency in predicting the LOS. However, as these models are based on existing data, it is understood that the more this data is available, the better the models are trained and consequently achieve significantly higher accuracy. Several large databases, such as Medical Information Mart for Intensive Care (MIMIC)-IV, electronic ICU (eICU), etc., have been used to predict LOS in ICU, providing ICU nurses and physicians with crucial information for improving resource management and preventing complications [10]. Such a prediction could guide them to better staff allocation strategies such as avoiding overload, especially during periods of high demand [12]. A tool with direct calculation of LOS could also help the ICU clinicians in balancing the phenomena of overstays, early discharges, readmissions, and dealing with increased rates of complications and patient mortality [11,13-16].

ML-based prediction models, despite their promising advantages, are rapidly developing but at the same time present many challenges. Biases in data collection and sample representativeness, validation and generalizability of results, interpretability, and complexity that often create suspicion among clinicians in predictions are some of the major challenges [8]. The weakness of prediction models for both LOS and mortality in the ICU is also due to the lack of large and multicenter datasets to establish a generalized model for all ICU patients [17-19]. Numerous approaches have been proposed both to overcome these issues [20] and achieving the highest possible forecast accuracy. Most existing models are still relying on single algorithms, which are often limited by the above issues and data bias.

Although ensemble learning has demonstrated enhanced predictive performance [21,22] and is of great utility, no research has yet applied stacked ensemble models to directly predict ICU LOS. Stacked ensemble models combine various machine learning algorithms, which improves overall prediction precision [23]. Even with this benefit, there is limited research on applying this method to predict ICU LOS. To address this gap, the present study aims to develop a stacked machine learning approach with a satisfactory prediction accuracy of adult patient LOS in ICU.

## Materials and methods

Dataset analysis

The dataset utilized in this study is obtained from the eICU Collaborative Research Database, including a diverse collection of more than 200,000 patient records from multiple ICUs across a range of hospitals in the USA, involving patients admitted to critical care units in 2014 and 2015. This allowed us to train our models using data from multiple data sources rather than a single ICU entity. That database enabled us to use specific features related to patient demographics and clinical measurements. 

As demonstrated in Table 1, the dataset consisted of 35 input features - 18 categorical and 17 numerical - and a numerical output representing the ICU patients’ LOS. These clinical characteristics were adopted from the APACHE IV prediction model. However, we decided not to incorporate all the features present in APACHE IV. We omitted a) LOS before ICU, and b) admission diagnosis, because these characteristics do not reflect the patients’ current clinical status. Although they are included in many LOS prediction models, they have been questioned for their actual contribution and limited real predictive value, as they are overshadowed by other factors. Our approach was based on the theory that ICU patient prediction models would have to prioritize variables that reflect real-time clinical status over pre-admission characteristics. The presumption is that features associated with the patients’ clinical decision do not adequately represent their current ICU status, as they are characterized by continuous variations that make static features of the prior diagnosis quickly outdated [24].

**Table 1:** Dataset variables

Category	Variables
Categorical	Age, Mechanical Ventilation, Glasgow Coma Scale (GCS) (Eyes, Verbal, and Motor response), Chronic Renal Failure or Hemodialysis (CRF/HD), Lymphoma, Cirrhosis, Leukemia, Hepatic Failure, Immuno-suppression, Metastatic Cancer, AIDS, Thrombolysis, Origin, Readmission, Emergency Surgery, Operative/Non-operative
Numerical	Temperature (°C), Mean Arterial Pressure (MAP) (mmHg), Heart Rate (HR) (bpm), Respiratory Rate (RR) (breaths/min), Fraction of Inspired Oxygen (FiO₂) (%), Arterial Partial Pressure of Oxygen (PaO₂) (mmHg), Arterial Partial Pressure of Carbon Dioxide (PaCO₂) (mmHg), Arterial pH, Sodium (Na⁺) (mmol/L), Urine Output (mL), Creatinine (mg/dL), Blood Urea Nitrogen (BUN) (mg/dL), Blood Sugar Level (BSL) (mg/dL), Albumin (g/dL), Bilirubin (mg/dL), Hematocrit (%), White Blood Cell Count (WBC) (cells/μL)
Output	Length of Stay (days)

Indicatively, there are about 116 individual variations of "admission diagnosis" in APACHE IV. A wide variety of these diagnoses have been well-documented and strongly associated with increased ICU LOS. However, there are also several diagnoses accompanied by contradictory studies that do not adequately confirm this association in the literature [25]. Therefore, given the uncertainty, we considered that this variable might not necessarily enhance the predictive value of our final models.

Prior to deploying any predictive modeling techniques, we proceeded with data preprocessing and data cleaning; these steps are necessary for the integrity and reliability of the dataset to be further used. Specifically, missing values handling, normalization, categorical features identification, and encoding were the main strategies applied for the preprocessing. That involved correcting inconsistencies and removing dataset records, like missing or null values in the outputs. Thus, the final dataset consisted of 148,532 patient records.

Methodology

For the purpose of this research, we included only the adult patients from the eICU database. We leveraged different machine learning algorithms with respect to our research objectives. We considered the algorithmic methodologies presented in the following paragraph to determine which models are most appropriate for providing precise LOS predictions. In addition, we employed ensemble learning, a method that combines predictions from multiple models to take advantage of their individual strengths, leading to improved results. 

For a better understanding and prediction delivery, the initial dataset was split into two groups based on the patients’ respective ICU LOS: short-term patients (actual LOS ≤ 7 days) and long-term patients (actual LOS > 7 days). This cutoff point is supported by clinical studies and evidence linking the LOS to the respective patient outcomes and resource use. Indicatively, several studies present and document the specific cutoff based on both clinical and methodological criteria [26-29]. Moreover, the studies by Weissman et al. [30] and Lefering et al. [31] identify this cutoff as an indicator for increased complications and resource utilization, respectively. Additionally, Zebin et al. [32] and Alsinglawi et al. [33] both employed the 7-day threshold in splitting ICU stays, confirming its utility in practice towards prediction and resource planning. The aforementioned threshold is commonly applied in machine learning research forecasting ICU LOS. According to this evidence, the original dataset was partitioned into two sets to study and evaluate the performance of the algorithms described below.

The decision trees algorithm was selected due to its simplicity and ease of interpretation, making it essential. Furthermore, methods such as Extra Trees and Random Forests were chosen; these utilize decision tree structures to enhance performance and provide analytical outcomes on feature importance without requiring complicated finetuning [34]. Modern algorithmic methodologies such as CatBoost, LightGBM, and XGBoost were also selected to be utilized for their proven ability to handle similar challenging prediction problems. The most evident benefit of CatBoost is that it supports categorical data very well; one of its noteworthy characteristics is that no traditional transformations are required before using the model, which decreases overall model complexity [35]. Moreover, LightGBM excels in training speeds and memory efficiency, particularly in large datasets, due to its histogram-based algorithm [36]. Additionally, XGBoost is preferred for its regularization techniques, preventing overfitting. That increases the model robustness in a way that ensures reliable performance in similar applications [37].

To determine the most suitable models, we applied first the default settings (hyperparameters) defined by each of the Python libraries so that the discrete algorithms' and frameworks' comparisons are conducted on fair ground. This gave us a high-level view before any kind of further manual tuning, following the recommendation of Bottou et al. [38].

Later, we applied each algorithm’s native preprocessing features where available, using the tools provided by each discrete algorithm. With these features integrated, we improved the models' performance. This step provided a standard baseline when comparing the models against each other with their default configurations for more accurate evaluations between the algorithms. To achieve the best performance measures for our model, we also performed hyperparameter tuning using random search. This method assigns random values to the aforementioned parameters during the model training, making it effective and time-efficient, especially in high-dimensional spaces [39]. That allowed us to explore various configurations and identify the optimal hyperparameters, achieving better results for our models. 

**Figure 1:**
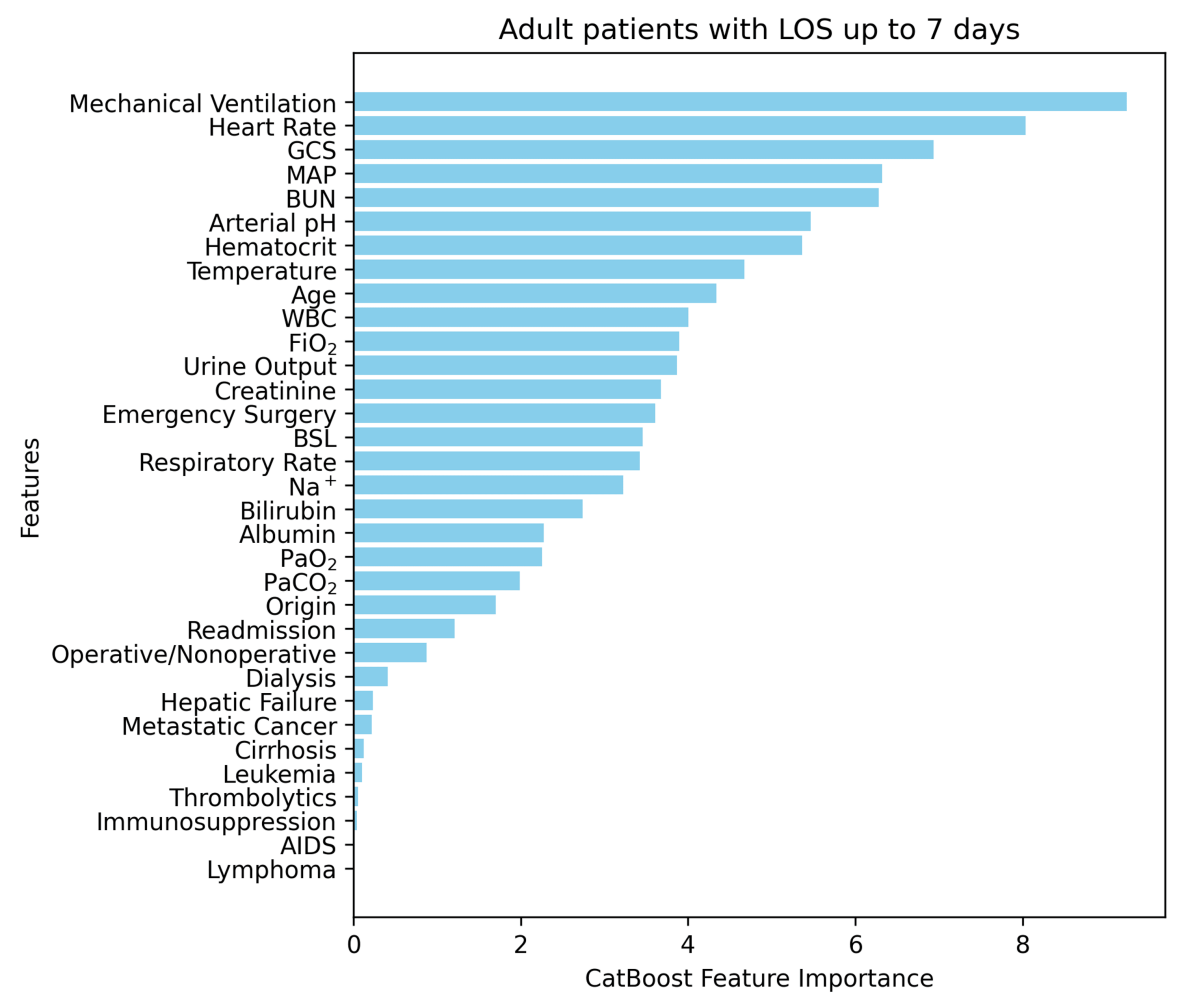
Feature importance analysis of the most dominant model for ICU LOS≤7 days. LOS: length of stay; GCS: Glasgow Coma Scale; MAP: mean arterial pressure; FiO₂: fraction of inspired oxygen; PaO₂: arterial partial pressure of oxygen; PaCO₂: arterial partial pressure of carbon dioxide; Na⁺: sodium; BUN: blood urea nitrogen; BSL: blood sugar level

**Figure 2:**
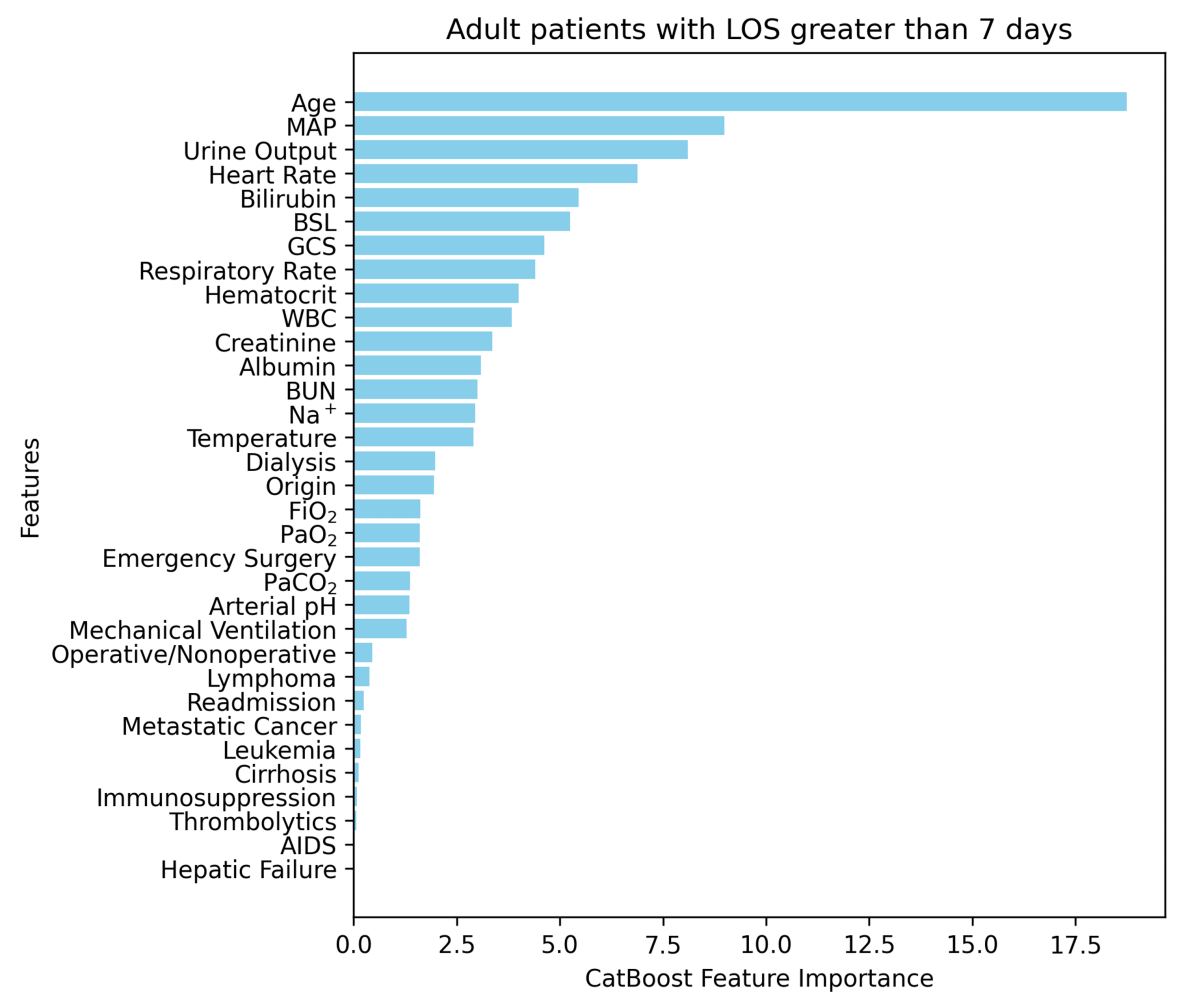
Feature importance analysis of the most dominant model for ICU LOS >7 days. LOS: length of stay; GCS: Glasgow Coma Scale; MAP: mean arterial pressure; FiO₂: fraction of inspired oxygen; PaO₂: arterial partial pressure of oxygen; PaCO₂: arterial partial pressure of carbon dioxide; Na⁺: sodium; BUN: blood urea nitrogen; BSL: blood sugar level

In the final form of our models, we implemented an additional feature engineering step that played a significant role in the improvement of the metrics. This allowed us to identify the most important input features for each discrete model architecture by running a pipeline of actions. First, we started with the initial model training, using their predefined baseline configurations as mentioned. Afterwards, the discrete features were evaluated and ranked based on their importance scores derived from the respective models. In this way, we could eliminate the least important feature set during the ranking process. This iterative process allowed us to retrain the respective models on the new selection of features, reducing their dimensionality. In that way, we could evaluate our final models against the test dataset, including data that were not known to the trained models by then. Indicatively, Figure 1 and Figure 2 depict the feature importance analysis conducted using the most important sub-models from the final models in both cases. 

To further improve our models’ performance, we used a method called ensemble learning. It utilizes the strengths of different algorithms, and by that, it becomes robust by reducing the error and enhancing the reliability of predictions [40]. Based on the aforementioned methods, we delivered one ensemble learning model for each group of patients, outperforming the individual ones. 

The criteria for selecting the best hyperparameters for each model were based on commonly accepted regression performance measurements: Root Mean Squared Error (RMSE), Mean Squared Error (MSE), and Mean Absolute Error (MAE). RMSE was our primary measurement of performance since it is penalized more for larger errors with its squared element. Furthermore, RMSE has the same unit as the data and hence is more interpretable; thus, the specific metric is more commonly used in similar studies. These metrics collectively give the measure of predictive accuracy and generalization ability of a model [41], which helps to gain better insight into its performance. 

For the ICU LOS of up to 7 days, the final ensemble learning structure consisted of two XGBoost and four CatBoost models. Each model was carefully selected and configured to ensure cohesive integration within the ensemble, maximizing overall performance. Specifically, the first XGBoost model used a slower learning rate of 0.075 with a higher minimum child weight of 5 and a max depth of 8, whereas the second one adopted a faster learning rate of 0.1 and a lower child weight of 1. Among the CatBoost models, two of them shared a learning rate of 0.1 and full feature sampling (random subspace method (RSM)=1) but had a different depth definition - 1 and 6. The remaining CatBoost models used a learning rate of 0.05 and an RSM of 0.8. Their architecture was identical, but they differed on the data they were trained on, as the latter one was based on a stacked learner, meaning that it was trained on the predictions of the prior model. Thus, it was faster in training and could deliver an improved RMSE metric.

Analogously, for patients with an ICU LOS greater than 7 days, we focused on capturing more complex relationships between the input features of the dataset, relating to longer stay periods. The stacked ensemble learning architecture was based on one CatBoost and four XGBoost models. The XGBoost models differ mainly in their hyperparameters, such as the learning rate, subsampling ratio, and feature sampling strategies. Two of these models have a maximum tree depth of 8, whereas the other two have a maximum depth of 7 and 8. Their learning rates are either 0.075 or 0.1, with two models linked to each value. This configuration delivers robust results, as the final model predictions are also enhanced by the CatBoost model that is trained with a learning rate of 0.05, a depth of 8, and an RSM equal to 0.8, allowing a randomized feature selection on every split, leading to a better model generalization. The final stacked ensemble learning model was specifically optimized for longer stays by achieving significantly improved accuracy over the individually defined ones for extended purposes as well, enhancing resource allocation and clinical decision-making.

For validation purposes, we employed a 10-fold cross-validation strategy throughout the training process of the aforementioned models. The total training time for the first stacked model (ICU LOS≤7 days) needed was 1438.22 seconds, whereas for the second one (ICU LOS >7 days) it was 1089.29 seconds, using an Apple M1 Max 32-Core GPU with 32 GB of unified RAM (Apple, Inc., Cupertino, USA). By following the aforementioned preprocessing method and fine-tuning the respective hyperparameters of each discrete model, we enhanced accuracy and delivered better results compared to our general model.

Ethical considerations

For this study, we received approval from PhysioNet to access the credentialed eICU Collaborative Research Database. One of the authors was authorized to use the restricted dataset, with access granted on March 14, 2024, at 10:24 a.m. Prior to approval, they completed the required training through the Collaborative Institutional Training Initiative (CITI Program), under Record ID 61757420.

## Results

**Figure 3:**
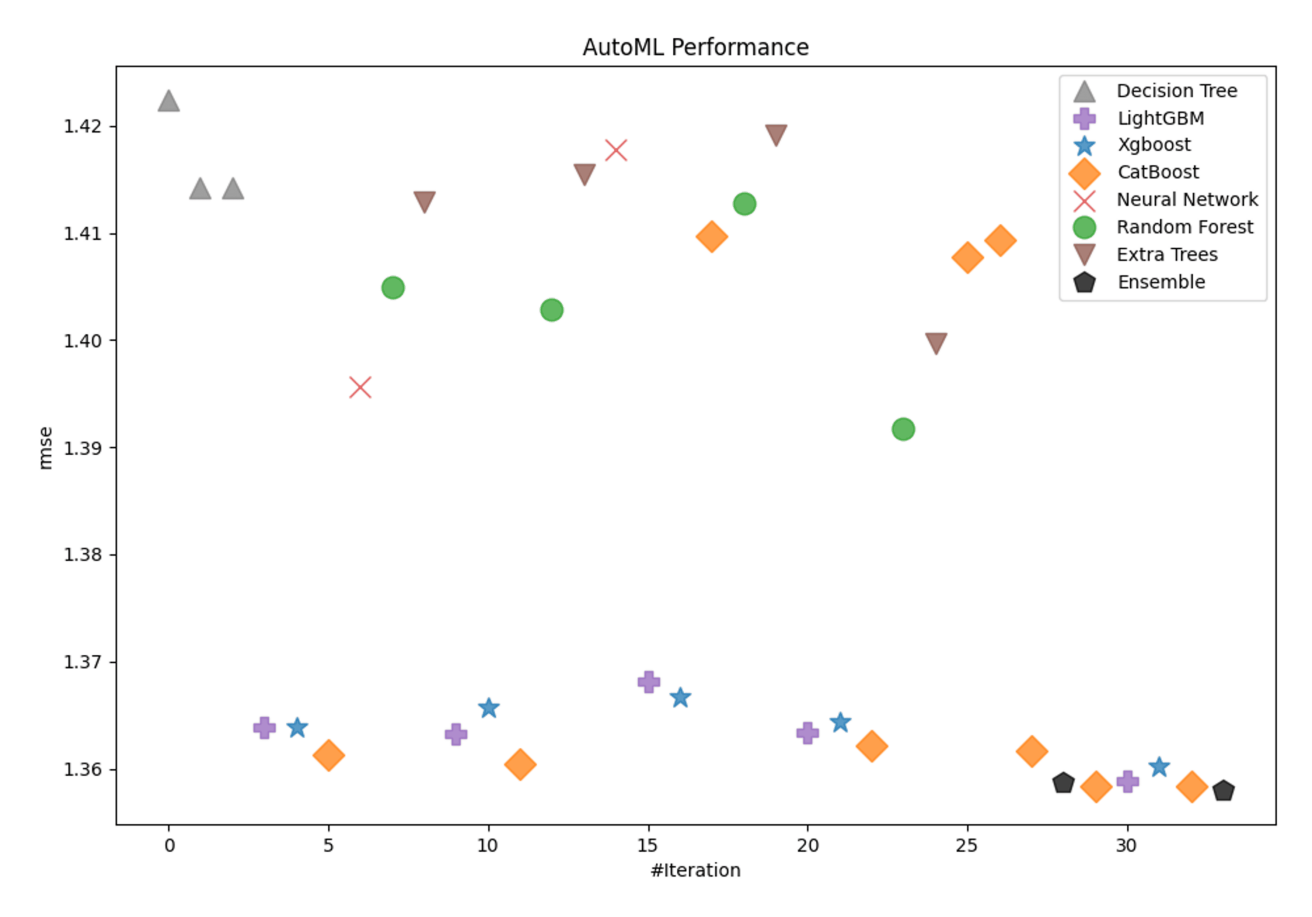
Model performance comparison for patients with ICU LOS ≤7 days. LOS: length of stay

**Figure 4:**
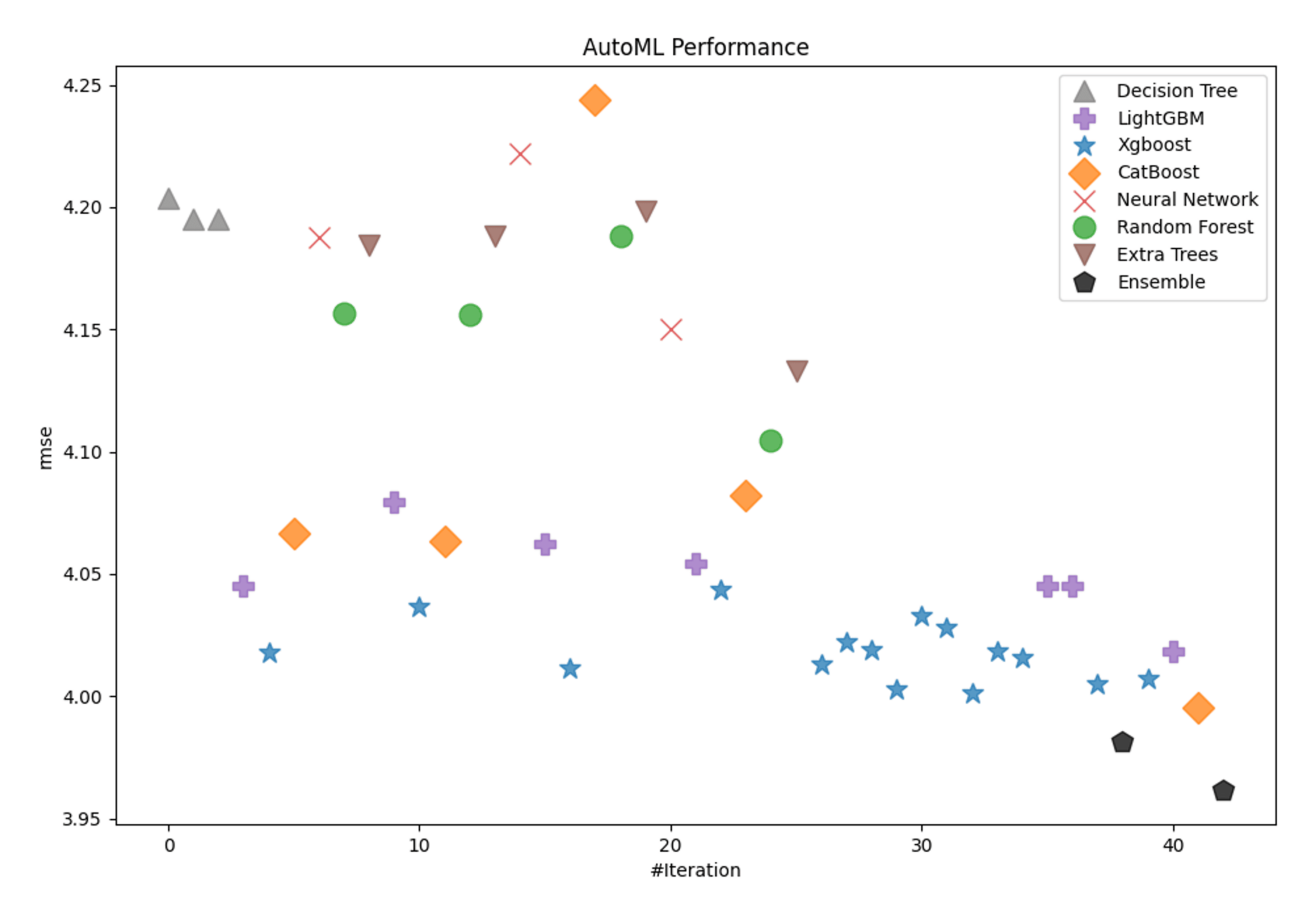
Model performance comparison for patients with ICU LOS >7 days. LOS: length of stay

This paper evaluated many machine learning algorithms to predict the Length of Stay in ICUs. In the current analysis, the following discrete algorithms were included: Decision Trees, Random Forests, Extra Trees, CatBoost, LightGBM, and XGBoost. Decision Trees were among the first models developed, being straightforward and easily interpretable; however, they had significant shortcomings in dealing with complex relationships in the input features of the dataset. Random Forests, an ensemble method based on decision trees, gave better results while improving the model's generalization capabilities, although it was still not able to deliver excellent metrics. However, improvements - although small in most cases - were present in all the metrics evaluated. In summary, Decision and Extra Trees, as well as Random Forests, returned the poorest performance among all the algorithms evaluated in this study. On the other hand, CatBoost, LightGBM, and XGBoost demonstrated similar levels of performance, resulting in promising results with closely matched metrics. Therefore, more modern models had to be developed to capture complex relationships among the input components. The overall performance of the models for both categories (short- and long-term patients) are depicted in Figure 3 and Figure 4.

**Figure 5:**
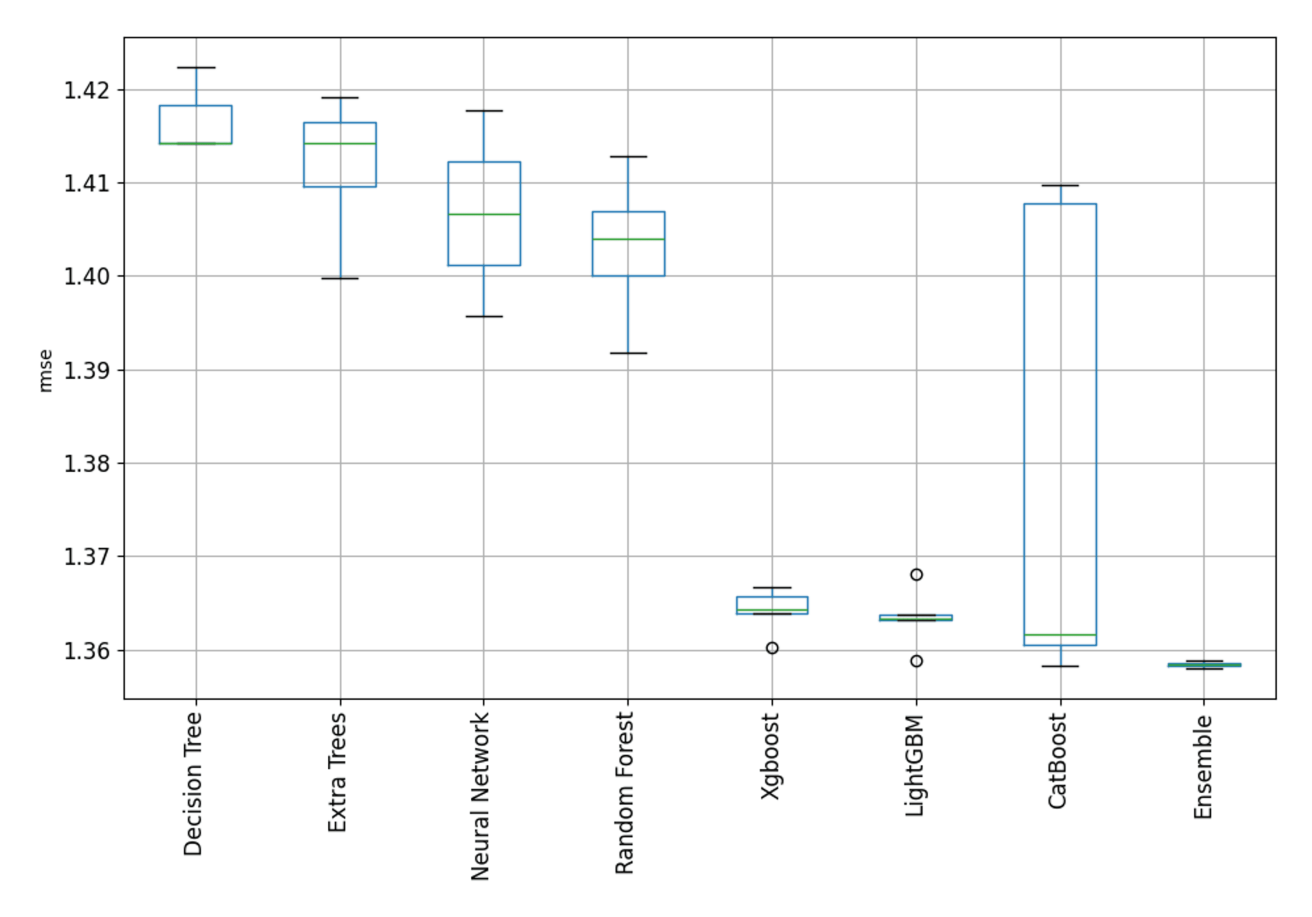
Boxplot comparisons of performance across model types for patients with ICU LOS ≤7 days. LOS: length of stay

**Figure 6:**
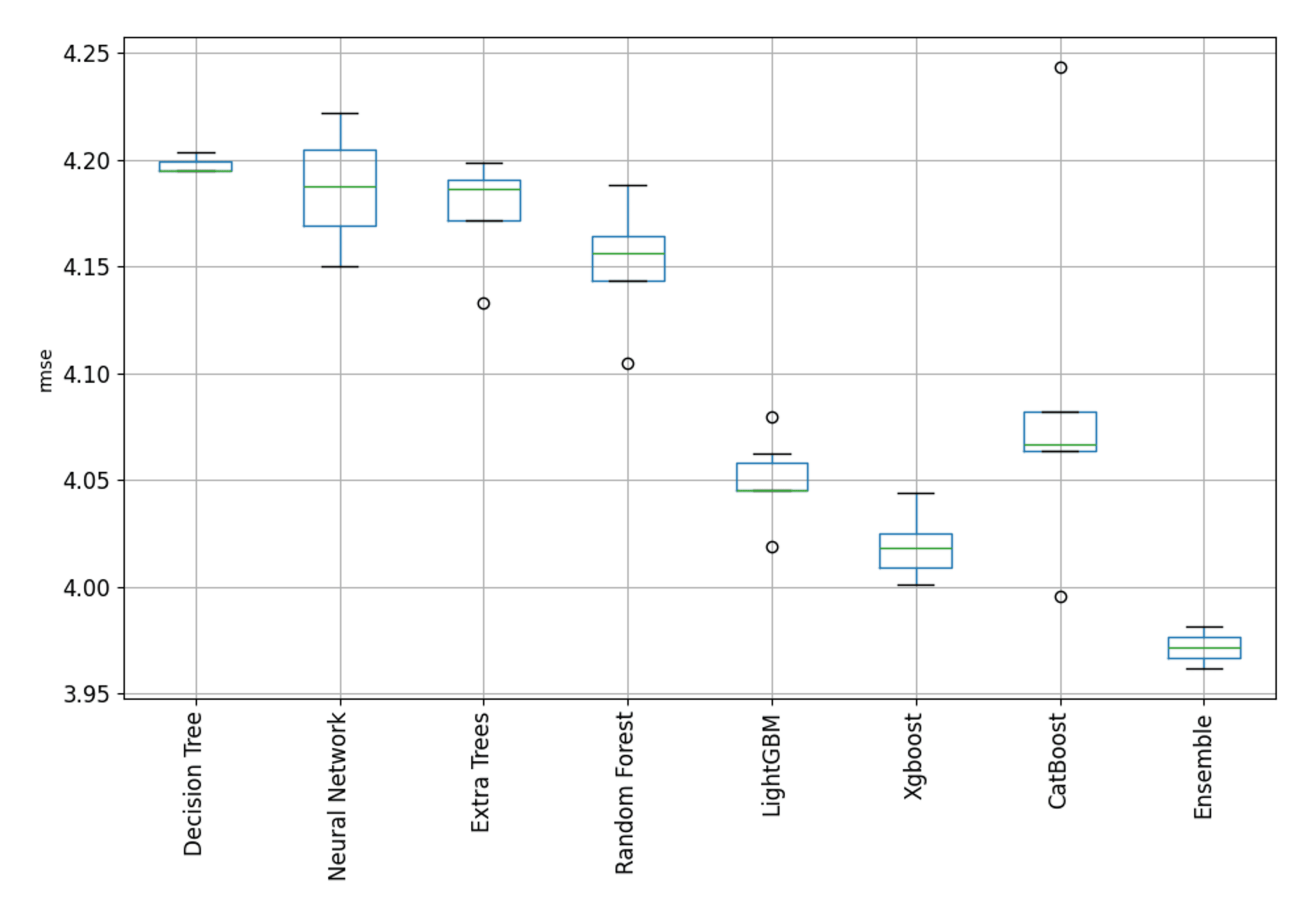
Boxplot comparisons of performance across model types for patients with ICU LOS >7 days. LOS: length of stay

In both cases, CatBoost, LightGBM, and XGBoost demonstrated similar levels of performance, resulting in promising results with closely matched metrics. As measured by the RMSE metrics, CatBoost delivered the lowest discrete-model RMSE for short-term patients at 1.358, with XGBoost following at 1.366. Similarly, for long-term patients, CatBoost achieved the lowest discrete-model RMSE at 3.9951, followed closely by XGBoost at 4.007. Figure 5 and Figure 6 show the distribution of the RMSE metric across the developed models grouped by type to provide a clear visualization and highlight the ensemble potential compared to single models. The boxplots show clearly the skewness and range of performances across the different model categories. Based on graphical analysis, XGBoost, LightGBM, and CatBoost were the most effective single model types. In the context of the current research, such visualization provides overall insights into the relative strengths and limitations inherent in each model type. Although the metrics of the last three algorithms were affordable, our ensemble learning model architecture provided better metrics overall. Some final indicative performance metrics for short-term patients (ICU LOS ≤7) were an RMSE of 1.358 and a MAE of 1.037. For long-term patients (ICU LOS>7), the final ensemble learning model delivered an RMSE of 3.96 and a MAE of 1.997. Overall, CatBoost, LightGBM, and XGBoost were the most dominant distinct models for predicting the LOS in an ICU because they work effectively with high-complexity data and deliver affordable prediction metrics, but the ensemble learning model was found to outperform them as a combination of strengths.

Table 2 presents a comprehensive overview of the distribution and the characteristics of each dataset, as well as their respective metrics delivered by their final ensemble models. Ultimately, the results below highlight the effectiveness of stacked ensemble learning, especially on challenging, high-complexity, and imbalanced datasets.

**Table 2:** Predictive indices based on actual ICU LOS results. LOS: length of stay; MAE: Mean Absolute Error; MSE: Mean Squared Error; RMSE: Root Mean Squared Error

Metric	N	Actual LOS		Predicted LOS			
	(%)	Mean (SD)	Median, IQR	MAE	MSE	RMSE	R^2^
≤7 days	135,397 (91.38)	2.064 (1.458)	1.7, 1.9	1.037	1.844	1.358	0.167
>7 days	12,762 (8.62)	12.991 (9.062)	10.6, 6.39	1.997	15.695	3.96	0.184

## Discussion

The aforementioned metrics of the current study are affordable compared to previous studies. According to the study from Alghatani et al. [21], their prediction of LOS in ICU achieved an MAE from 2.81 to 3.51 and an RMSE of ≃ 6.03. The mean and the median of actual LOS were approximately 4.75 and 2.64 days, respectively. In a total of 1592 ICU heart failure patients from the MIMIC-III database, Alsinglawi et al. [33] showed the Gradient Boosting Regressor (GBR) as the most efficient model with R² = 0.84 and MAE = 2.00 after hyperparameter optimization. The actual LOS of patients was on average 10.77 days, with a median of 8.13 days. Similarly, in the study by Chen et al. [42] from a large sample from the MIMIC-IV database, the results showed R2 = 0.57, RMSE = 3.61, and MAE = 2.42 days. Cuadrado et al. [11] reported a “Day to Discharge” model with different prediction evaluations based on different target days. For example, the accuracy for short stays (≤7 days) showed RMSE ≈ 0.7, while for long stays (≥15 days) the RMSE reached up to 6.5 days (errors increased exponentially as they moved away from the day of discharge). 

In another interesting attempt, researchers with a sample from the same database (MIMIC-IV) used a combination of LOS prediction. They first classified patients based on predicting low (≤4) or high (>5) LOS and then focused on predicting LOS with regression. XGBoost delivered the most promising results for the group of patients with small actual LOS (≤ 4 days): MAE = 1.99, R² = 0.19. At larger actual LOS (patients with 5 and 10 days), the results showed MAE ≃1.5 and ≃6.5 days, respectively [1]. 

Similar notable efforts have been applied by many authors, but several challenges remain crucial in the analysis. For example, some authors document the selection of features based on some basic clinical significance [21], while others admit that they omit some important comorbidity characteristics mainly for reasons of economic or ease of measurement [33]. The results, however, are not always clear, and further research is required. For example, in the Verburg et al. study [43] authors found statistically significant associations between ICU LOS and certain unit characteristics. However, incorporating these characteristics into regression models for predicting LOS did not significantly improve the accuracy, compared with models based on patient characteristics alone. A more reliable LOS prediction tool could clarify many of these associations and enhance research by identifying - or excluding - factors that influence ICU patient LOS.

Furthermore, in most cases of related research, the approaches focus more on the technical and mathematical aspects, and often, a systematic methodology for the initial selection or justification of the features that emerge as the most clinically significant is omitted. Moreover, the correlation of findings with existing clinical guidelines or studies that support their selection or the importance of the variables from a clinical perspective is rare [33]. Several authors also do not include key metrics in results, such as RMSE, or measures of dispersion and variance of actual LOS. Thus, the evaluation of models’ relative performance (how "large" an error is relative to the actual dispersion of values in the population) is difficult [11,21,33].

Most studies evaluate model performance using metrics such as MAE, RMSE, and R², comparing the results with those of similar models from previous studies [12,21,44]. However, the international literature does not provide a clearly defined threshold for what constitutes a “clinically acceptable” prediction error regarding ICU LOS. In a relative review, an MAE of less than one day, in populations with a mean LOS of 5-8 days, is characterized as a “particularly useful” prediction, especially for objectives such as optimal staff and resource planning [11]. The most advanced prediction models for ICU LOS typically report MAE values between 1 and 2 days, and MAE <1 day when the prediction specifically targets the final stages of hospitalization (i.e., the last week before discharge) [11,45]. For instance, Rocheteau et al., using a temporal pointwise convolution model, have achieved an MAE of approximately 1.55 days on the eICU dataset and 2.28 days on the MIMIC-IV dataset [45]. In another review, it is reported that MAE and RMSE values in LOS prediction models vary considerably, with typical MAE values ranging from 2 to 3 days and RMSE values from 3 to 7 days [46]. Overall, although modern methods achieve MAE between 1 and approximately 2 days, or even less in specialized applications, what is considered a clinically acceptable margin of error or level of accuracy has not yet been clearly defined in international literature. This highlights the need for further research to establish standardized criteria for the comparability of results.

Another controversial point is the exclusion of non-survivors. This makes sense, as it gives an advantage to the model, making the dataset more homogeneous and therefore resulting in better accuracy and performance (lower RMSE/MAE error values). On the other hand, this exclusion seems to be inappropriate for ICU resource planning as it may underestimate the resource use needs. Moreover, exclusion of non-survivors makes prediction difficult to implement in real-time, unless combined with mortality prediction. Finally, it may be inaccurate because of false optimistic predictions of patients who will eventually die [11].

This study introduces a tool designed to be useful for clinical use, especially in decision making, efficient resource management, and quality of care improvement. Physicians and nurses with a highly accurate prognosis as a tool can plan more efficiently the patients’ care, especially those with a high likelihood of a prolonged ICU stay. Long ICU stays are often associated with deterioration of the patient's condition and often lead to futile care [47]. Optimal allocation of resources, such as staff, equipment, or beds, is very useful, especially in times of high pressure [12,43]. Knowledge of the likely length of hospital stay can also be beneficial to physicians, as it enables them to identify high-risk patients and implement timely interventions to prevent complications such as infections, multi-organ failure, and sepsis [17,48,49]. Moreover, especially for nurses, the consistent implementation of established protocols facilitates autonomous clinical decision-making, as noted by Bani Hamad et al. [18]. LOS is a key indicator for evaluating the effectiveness and quality of care provided in ICUs and helps identify areas requiring improvement [7]. Studies have documented a correlation between nursing-sensitive quality indicators and LOS in adult intensive care units [50]. Adequate staffing, an optimal nurse-to-patient ratio [43], and interventions focusing on patient-centered or palliative care are all associated with reduced LOS [51-53]. Furthermore, the ability to predict LOS enables nurses to inform families accurately and promptly about the expected duration of hospitalization, thereby enhancing communication and building trust [48,49]. Conversely, increased nursing workload, higher disease severity, and a greater number of nursing diagnoses are linked to prolonged hospitalization, highlighting the complexity of nursing care as a determining factor for LOS [54,55].

Stacked ensemble over individual models in ICU LOS Prediction

In the ensemble learning method, accuracy appears to improve, outperforming other contemporary techniques [56]. For example, in the study of Naseem et al. [57], an impressive accuracy of 98.83% was achieved in breast cancer diagnosis and prognosis. In another study, ensemble bagging and boosting techniques improved the prediction of heart disease risk by 7% [58]. In our study, the stacked ensemble learning approach achieved an MAE of 1.037 and 1.997 for ≤7 and >7 days, respectively. 

An MAE of about 1 day suggests that, on average, the model's predictions are reasonably accurate. With a prediction error of about one day, it is feasible to anticipate and limit potential unexpected events in daily planning or to identify at-risk patients early (e.g., those susceptible to infection or difficulties from mechanical breathing) for more prompt and focused intervention. The difference in MAE on predictive value varies between the dataset subsets. For those having an actual LOS of less than 2 days (median = 1.7), a 1-day error represents a 60% deviation. For those having an actual LOS of about 10 days (median = 10.6), the same measure of error gives a deviation of 9%. This gap reflects the model's better performance for patients with long stays than those with short stays. 

This deviation difference may be accounted for by the instability of the patient during the early days after admission. In this period, critical care patients usually have acute and unpredictable changes in physiological variables. Unexpected complications or rapid deterioration are not necessarily fully captured in the traditional model variables, making prediction more difficult [23,59]. In addition, other non-clinical factors may lead to early discharge decisions that are often not recorded in medical records. Availability of resources or beds, non-strict admission criteria, futile care, hospital administrative policies, and new admission urgency in the ICU are some of such factors [60,61]. These variables may not easily be quantified and used in predictive models, leading to more complex data sets and inaccurate predictions of LOS, particularly for short-stay patients.

Clinical significance of the main features

In our models, feature importance analysis was derived from CatBoost, the most dominant model. The five most significant features for the short-term group were Mechanical Ventilation (MV), Heart Rate, Glasgow Coma Scale (GCS), Mean Arterial Pressure (MAP), and Blood Urea Nitrogen (BUN), while for the long-term group, they were Age, MAP, Urine Output, Heart Rate, and Bilirubin. The aforementioned results are consistent with the literature. Mechanical ventilation has been associated with LOS by many authors. 

Peres et al. [25] in their meta-analysis found a 92% significance of MV, with 11 out of 12 studies demonstrating a positive association of MV with LOS. GCS score has also been well documented to be associated with ICU LOS, and in our study was a significant feature in both subgroups. However, as the GCS score is usually implicated in the decision to intubate, it seems that the relationship of GCS with ICU LOS is more complex. For instance, Hatchimonji et al. [62] showed that intubating patients with a GCS of 6 to 8 on arrival to the ICU was associated with a 13.7% increase in ICU LOS, with a mean duration of 5.5 days vs 4.8 days in the non-intubated (p<0.001). Analogously, other studies found no significant difference in ICU LOS among patients with GCS ≤5 [63]. Although there is insufficient evidence to use it as an independent predictor of ICU LOS, the relationship of GCS to required intubation is often studied and demonstrated. 

Age was the factor that emerged as the characteristic with the highest significance in the group of patients with a long stay (>7 days). Studies are often controversial, and their indirect positive relationship of Age in LOS is based rather on intermediate factors such as disease severity, comorbidities, and specific medical interventions [25,29,64-67]. 

Urine output and BUN are both related to kidney function and usually indicate possible underlying chronic diseases or serious health conditions. The meta-analysis by Søvik et al. [68] in 24 studies, including ICU trauma patients, found that patients with acute kidney injury had 4.0 to 7.9 days longer LOS. High total bilirubin levels have been positively associated with prolonged ICU stay in Urology and Nephrology patients [69] and an independent prognostic factor in patients with malignancies [70]. 

The variability of the heart rate has been associated with sepsis and multiple organ dysfunctions, inflammatory conditions, and may predict arrhythmias, death in cardiac patients, and increased intracranial pressure or brain death in neurological patients [71]. Numerous studies have reported that changes in vital signs occur several hours prior to a serious adverse event [72]. Heart rate has been associated with higher rates of mortality [73], and the recent data suggest that its integration into ICU scoring systems may lead to improved prognostic performance [74].

Limitations

Some limitations should be considered. First, the data set was from ICUs in the United States, which may limit the generalizability of our findings to other patient populations or different clinical practices. Second, the selection of input features was limited to those available in the eICU database, and some others were not considered intentionally. A different combination of inputs may affect the predictive performance of our model. Finally, external validation was not performed due to the unavailability of suitable external datasets. Until future studies address this issue, it represents a limitation for the generalizability of our model.

All aforementioned limitations must be taken into account when considering our findings. Future prospective and multicenter studies could help determine whether our model could be validated in a variety of clinical environments and in everyday medical practice.

## Conclusions

We tested a stacked machine learning approach that can mainly assist nurses in their decision-making. The model demonstrated a better ability to predict LOS in the ICU compared to individual models. We used advanced algorithms such as CatBoost, LightGBM, and XGBoost on a multicenter dataset with satisfactory results, especially in patients with long actual ICU stays. However, challenges remain, such as the relatively inferior performance metrics observed in patients with short actual ICU stays. Future development of the model could be oriented towards the models’ technical improvement and further validation for successful implementation in clinical practice.
